# MitoQ inhibits hepatic stellate cell activation and liver fibrosis by enhancing PINK1/parkin-mediated mitophagy

**DOI:** 10.1515/med-2021-0394

**Published:** 2021-11-12

**Authors:** Shi-Ying Dou, Jiu-Na Zhang, Xiao-Li Xie, Ting Liu, Jun-Li Hu, Xiao-Yu Jiang, Miao-Miao Wang, Hui-Qing Jiang

**Affiliations:** Department of Gastroenterology, The Second Hospital of Hebei Medical University, Hebei Key Laboratory of Gastroenterology, Hebei Institute of Gastroenterology, Hebei Clinical Research Center for Digestive Disease, Hebei, China; Department of Infectious Diseases, The Second Hospital of Hebei Medical University, Shijiazhuang, Hebei, China; Emergency Department, The Second Hospital of Hebei Medical University, Shijiazhuang, Hebei, China

**Keywords:** liver fibrosis, hepatic stellate cell, ubiquinone, PINK1 mitophagy

## Abstract

Mitophagy affects the activation of hepatic stellate cells (HSCs). Mitochondria-targeted ubiquinone (MitoQ) is a mitochondria-targeted antioxidant that reduces the production of intracellular reactive oxygen species (ROS). However, its relationship with mitophagy remains unclear. This study evaluated mitophagy during HSC activation and the effects of MitoQ on mitophagy in cell culture and in an animal model of the activation of HSCs. We found that MitoQ reduced the activation of HSCs and alleviated hepatic fibrosis. PINK1 (PTEN-induced putative kinase 1) is a putative serine/threonine kinase located in the mitochondria’s outer membrane. While the activation of primary HSCs or LX-2 cells was associated with reduced PINK1/parkin-mediated mitophagy, MitoQ reduced intracellular ROS levels, enhanced PINK1/parkin-mediated mitophagy, and inhibited the activation of HSCs. After knocking down the key mitophagy-related protein, PINK1, in LX-2 cells to block mitophagy, MitoQ intervention failed to inhibit HSC activation. Our results showed that MitoQ inhibited the activation of HSCs and alleviated hepatic fibrosis by enhancing PINK1/parkin-mediated mitophagy.

## 1 Introduction

Hepatic fibrosis is a common scarring response to chronic liver injury caused by various etiological factors, including viral hepatitis, alcohol abuse, autoimmune hepatitis, and nonalcoholic steatohepatitis. The degree of fibrosis is measured by semiquantitative scoring, such as the Ishak score [1]. Activation of hepatic stellate cells (HSCs) is a key step in the development of hepatic fibrosis. Resident HSCs become fibrogenic myofibroblast when they are activated, expressing α-SMA and producing large amounts of extracellular matrix proteins, such as collagen 1, leading to hepatic fibrosis. HSCs are activated by a variety of growth factors and cytokines, including transforming growth factor-β (TGF-β) [2,3]. Thus, inhibiting HSC activation could be an effective strategy for treating liver fibrosis.

Mitophagy is regulated by various factors. PINK1 (PTEN-induced putative kinase 1) is a putative serine/threonine kinase located in the mitochondria’s outer membrane. The PINK1/parkin pathway is considered the most important regulatory pathway, critical for the maintenance of mitochondrial integrity and function [4]. When a mitochondrial injury occurs, PINK1 recruits parkin to the mitochondria by phosphorylating both parkin and ubiquitin. This results in the activation of parkin’s E3 ligase activity and ubiquitination of mitochondrial outer membrane proteins (e.g., TOM20 and Mfn2), which eventually leads to degradation of mitochondrial proteins and clearance of damaged mitochondria via mitophagy [5,6,7]. Studies have shown that PINK1/parkin-mediated mitophagy plays a role in various diseases, such as nervous system [8], cardiovascular system [9,10], renal system [11], and liver diseases [12]. Specifically, mitophagy plays a role in hepatic fibrosis. Namely, PINK1/parkin-mediated mitophagy is enhanced in HSCs in a hepatic fibrosis-reversal model, and the inhibition of mitophagy enhances the activation of HSCs in mice [13].

MitoQ is a mitochondria-targeted antioxidant, which contains coenzyme Q10 and triphenyl phosphonium cations to allow it to enter the mitochondria and aggregate under the action of an electrochemical gradient, to reduce the generation of lipid peroxidation free radicals in the mitochondria, and to prevent lipid peroxidation [14]. MitoQ exerts a protective function in many diseases, such as cardiovascular disease, neurodegeneration, and liver fibrosis [14,15,16]. Mitochondria are the main organelle that produces reactive oxygen species (ROS). A number of studies have shown the relationship between MitoQ and ROS. MitoQ alleviates liver damage by reducing ROS production [17,18]. Several studies have shown the correlation between intracellular ROS level and mitophagy [19,20,21]. Increased ROS levels stimulate mitophagy and induce cell death. While inhibition of mitophagy increases ROS levels [22], enhanced mitophagy eliminates intracellular oxidative stress and reduces ROS production [23]. MitoQ regulates ROS production. However, it is still unclear whether MitoQ regulates mitophagy. A previous study has shown that MitoQ enhances mitophagy through the nuclear factor erythroid 2-related factor 2 (Nrf2)/PINK1 pathway and inhibits renal tubular epithelial-mesenchymal transition [24]. There are scarce data on MitoQ and PINK1/parkin-mediated mitophagy in the process of liver fibrosis. It is unclear whether MitoQ inhibits HSC activation by regulating PINK1/parkin-mediated mitophagy. Therefore, this study explored the relationship between MitoQ and PINK1/parkin-mediated mitophagy during HSC activation and the regulatory mechanism.

## 2 Materials and methods

### 2.1 Design of animal experiments

Thirty-six healthy male C57BL/6 mice 4–6 weeks old were purchased from Beijing Vital River Laboratory Animal Technology (Beijing, China), license number: SCXJ (Hebei Province) [1403088]. The animal experiments were conducted in the Laboratory Animal Center of Hebei Medical University. Animals were housed in a temperature- and humidity-controlled facility with standard laboratory chow and ad libitum access to food and water. Hepatic fibrosis was induced using carbon tetrachloride (CCl4) [14]. These animals were randomly divided into three groups: control (Oil), model (CCl4), and intervention (CCl4 + MitoQ) groups (*n* = 12 animals per group). The control group was given an intraperitoneal injection of olive oil at a dose of 5 mL/kg twice a week and 5 mL/kg olive oil intragastrically once every other day for 6 weeks. The model group was intraperitoneally injected with 5 mL/kg 10% CCl4 olive oil solution (CCl4: olive oil = 1:9, v/v) twice a week to induce hepatic fibrosis and also received 5 mL/kg olive oil intragastrically once every other day for 6 weeks. The intervention group was intraperitoneally injected with 5 mL/kg 10% CCl4 olive oil solution twice a week and also received 5 mL/kg olive oil containing MitoQ (10 mg/kg; MitoQ from Sigma-Aldrich, St. Louis, MO, USA) intragastrically once every other day for 6 weeks [18]. The animals were sacrificed 3 days after the final injection. All animals were anesthetized by intraperitoneal injection of phenobarbital (50 mg/kg), and their sera and livers were harvested. The animals were sacrificed by exsanguination, heartbeats were assessed to determine death, and adequate humanitarian care was given.

### 2.2 Serum aminotransferase activity

Serum alanine aminotransferase (ALT) and aspartate aminotransferase (AST) activities were determined by ALT and AST assay kits in accordance with the manufacturer’s protocol (Changchun Huili Biotechnology Co., Ltd., China).

### 2.3 Isolation and culture of primary HSCs

HSCs were isolated from C57BL/6 mice as previously described [25,26]. Briefly, the portal vein was cannulated with a 26-gauge needle. The liver was first perfused with an EGTA solution (Sigma-Aldrich) and then with collagenase and pronase (Gibco BRL, Gathersburg, MD, USA). The primary HSCs were isolated by Percoll density gradient centrifugation and, subsequently, cultured in Dulbecco’s modified Eagle medium/Nutrient Mixture F-12 (DMEM/F-12; Gibco, Carlsbad, CA, USA) supplemented with 20% fetal bovine serum (FBS; Life Technologies, Carlsbad, CA, USA) and cultured at 37°C in a humidified atmosphere with 5% CO_2_ for 1 day. The HSCs were identified by their property that newly isolated HSCs emitted blue-green fluorescence when excited by the ultraviolet light of 328 nm, which decreased within a few seconds. After 24 h of incubation, the medium was replaced with DMEM/F-12 supplemented with 10% FBS; at this time, the cell debris and nonadherent cells were removed by washing, and HSCs culture was 90% pure. After 3 days in culture, the cells were harvested for the subsequent experiments.

### 2.4 Morphology and immunohistochemical analyses of liver tissues

The livers were fixed with 4% paraformaldehyde, followed by embedding in paraffin, and sliced into 5-μm sections. Next, the sections were stained with H&E for morphological evaluation. Quantification of inflammation and necrosis was performed by a “blinded” liver pathologist, using a scale from 0 to 3 [27]. Immunohistochemical staining for α-SMA was performed as previously described [28]. Briefly, after deparaffinization, sodium citrate buffer was used for antigen retrieval. Subsequently, the slides were immersed in 3% hydrogen peroxide solution for 10 min, followed by incubation with 10% goat serum for 10 min. The sections were incubated with α-SMA antibodies (Cat. no. CY5295, 1:200, Shanghai Abways Biotechnology Co., Ltd., China) at 4°C overnight. After that, the slides were washed with PBS, incubated with secondary antibody (#SP-9001; Beijing Zhongshan Jinqiao Biotechnology Co., Ltd., China) and DAB chromogen (#ZLI-9018; Beijing Zhongshan Jinqiao Biotechnology Co., Ltd., China), stained with hematoxylin, and sealed. Staining intensities were quantified by measuring the positive area using ImageJ software. Masson staining was used for the assessment of collagen. Collagen deposition was quantified by measuring the positive area using ImageJ software. The degree of fibrosis was measured by semiquantitative scoring – the Ishak score [1].

### 2.5 Cell cultures

LX-2 Human Hepatic Stellate Cell Line (Cat. no. SCC064) was purchased from Merck. The LX-2 was cultured in DMEM (Gibco BRL, Rockville, MD, USA) containing 10% FBS in an incubator containing 5% CO_2_ at 37°C.

### 2.6 Measurement of intracellular ROS levels

Intracellular ROS levels were determined using a ROS Assay Kit (#S0033S, Beyotime, China). LX-2 cells from each treatment group were incubated in DMEM mixed with 10 μM DCFH-DA for 30 min. Next, they were washed three times in DMEM. The fluorescence was quantified using a fluorescence microplate reader with excitation and emission settings at 488 and 525 nm, respectively (BioTek, Biotek Winooski, Vermont, USA).

### 2.7 Western blot analysis

The primary HSCs and human LX-2 cells were treated with RIPA buffer (Sigma-Aldrich) containing 1 mmol/L phenylmethanesulfonyl fluoride (PMSF: Sigma-Aldrich), vertexed, and centrifuged to collect the supernatant. The protein concentration in the supernatant was determined using the Bradford assay. The western blot analysis was performed as previously described [29]. The following primary antibodies were used in this study: rabbit anti-α-SMA (Cat. no. CY5295, 1:1,000, Abways), rabbit anti-collagen 1 (Cat. no. AF7001, 1:300, Affinity Biosciences, OH, USA), rabbit anti-PINK1 (Cat. no. DF7742, 1:500, Affinity), rabbit anti-parkin (Cat. no. sc-32282, 1:1,000, Santa Cruz Biotechnology, Santa Cruz, CA, USA), rabbit anti-P-parkin (Cat. no. AF3500, 1:500, Affinity), rabbit anti-TOM20 (Cat. no. ab186735, 1:1,000, Abcam, Cambridge, MA, USA), and GAPDH (Cat. no. AF7021, 1:5,000, Affinity). The immunoreactive bands were visualized using an Odyssey infrared imaging system (LI-COR Biosciences, Nebraska, USA). For protein quantification, the bands were scanned and quantified using Image-Pro Plus 6.0 software (Datacell, UK).

### 2.8 siRNAs transfection

The small-interfering RNAs (siRNAs) were synthesized by Gene Pharma (Shanghai China). and included the following sequences for PINK1: 5′-GGA GCA GUC ACU UAC AGA ATT-3′and 5′-UUC UGU AAG UGA CUG CUC CTT-3′. The negative control (NC) sequences were 5′-UUC UCC GAA CGU GUC ACG UTT-3′ and 5′-ACG UGA CAC GUU CGG AGA ATT-3′. Briefly, when the LX-2 cells were 60% confluent in the plate, we added the diluted siRNA to diluted RNAiMAX reagent (Thermo Fisher Scientific, Waltham, MA, USA) and incubated for 5 minutes at room temperature. Subsequently, added to cells and incubate for 6 h at 37°C. After that, we replaced the serum-free DMEM with DMEM containing 10% FBS and incubate it for 24 h. Then, the cells were divided into the following treatment groups: si-NC, si-NC + MitoQ (MitoQ 2 ng/mL), si-PINK1, and si-PINK1 + MitoQ (MitoQ 2 ng/mL). Every group incubated 24 h with serum-free DMEM to synchronization then MitoQ was added and incubated for 24 h.

### 2.9 Statistical analysis

The measurement data are presented as mean ± standard deviation (*x* ± SD). SPSS 16.0 software (IBM SPSS Inc., Chicago, IL) was used for statistical analysis. Differences of means between multiple groups were compared by one-way ANOVA and the least significant difference (LSD) test. Differences of *P* < 0.05 were considered statistically significant.


**Ethical approval:** The research related to animal use has complied with all the relevant national regulations and institutional policies for the care and use of animals (2021-AE003).

## 3 Results

### 3.1 MitoQ inhibits mouse liver fibrosis

**Figure 1:**
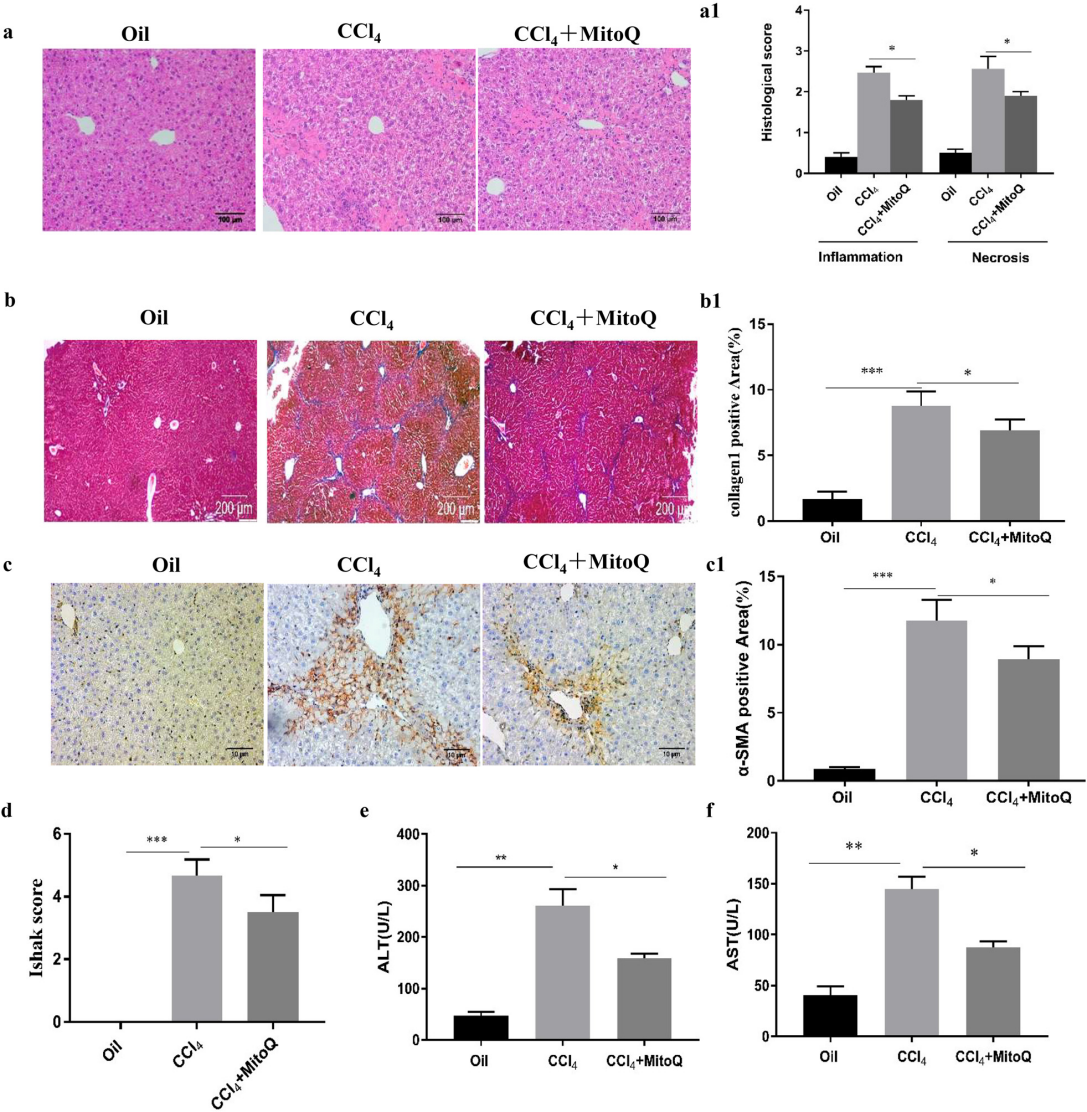
MitoQ inhibits mouse hepatic fibrosis. (a) Representative images of hematoxylin and eosin (HE) staining of liver tissue (100× magnification). (a1) Quantification of liver inflammation and injury was performed. Results are presented as mean ± standard deviation. *P < 0.05 indicates significant differences relative to the indicated groups; (*n* = 6). (b) Masson’s trichrome staining of the liver tissue (40× magnification); (*n* = 6). (b1) Columns represent the percentage of positive area of collagen fibers. Results are presented as mean ± standard deviation. *P < 0.05, ***P < 0.001 (*n* = 6). (c) Immunohistochemistry for α-SMA in the liver tissue (100× magnification). (c1) Columns represent the percentage of α-SMA positive area. Results are presented as mean ± standard deviation. *P < 0.05, ***P < 0.001 (*n* = 6). (d) Liver fibrosis semiquantitative scoring was performed. *P < 0.05, ***P < 0.001; (*n* = 6). (e) Serum ALT levels. *P < 0.05, **P < 0.01 (*n* = 6). (f) Serum AST levels. *P < 0.05, **P < 0.01 (*n* = 6).

To evaluate the effects of MitoQ on hepatic fibrosis, we established a model of CCL4-induced hepatic fibrosis [13]. Hepatic damage was assessed by morphological analysis of H&E-stained slides. Fibrosis degree was assessed by Masson’s trichrome staining and immunohistochemical staining for α-SMA. The liver sections showed inflammation and necrosis in the model group, which was alleviated by MitoQ intervention (Figure 1a and a1). Masson’s trichrome staining showed significant proliferation and deposition of the collagen fibers in the model group, which was reverted after MitoQ intervention (Figure 1b and b1). Results of α-SMA immunohistochemistry showed increased α-SMA expression in the model group, whereas MitoQ intervention significantly reduced the α-SMA expression (Figure 1c and c1). The Ishak scores of the mice injected with CCl_4_ were significantly higher than those of the oil-treated mice. However, after treatment with MitoQ, the CCl_4_-injected mice had significantly reduced Ishak scores (Figure 1d), suggesting that MitoQ intervention inhibited collagen secretion in mouse livers with fibrosis. Serum ALT and AST levels were significantly higher in the model group than in the oil group. MitoQ intervention significantly reduced the ALT and AST levels (Figure 1e and f). These findings showed that MitoQ alleviated liver damage and liver fibrosis.

### 3.2 Mitophagy decreases with the activation of stellate cells, and MitoQ enhances mitophagy and reduces ROS production

**Figure 2:**
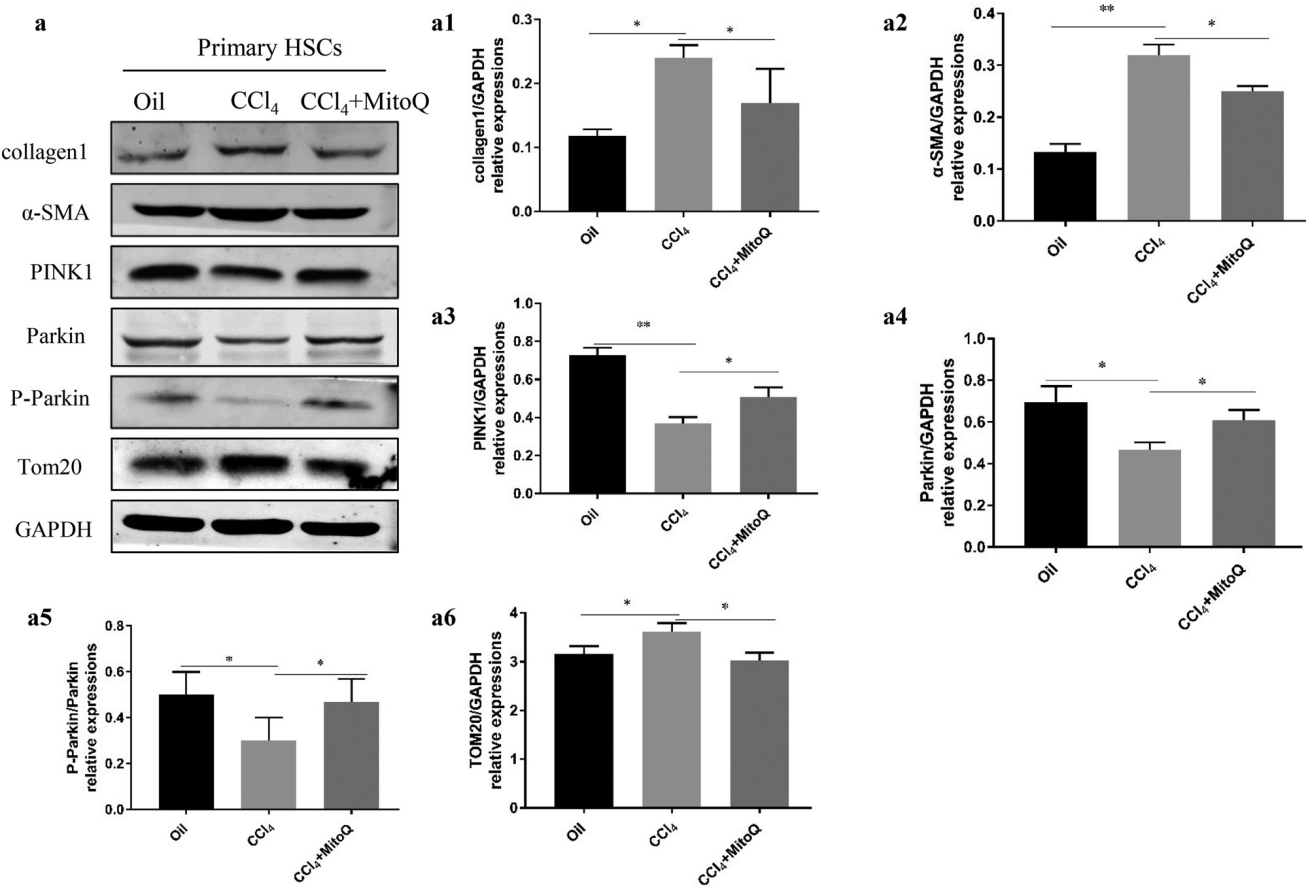
Mitophagy decreases with the activation of HSCs, and MitoQ enhances mitophagy. (a) Western blot analysis shows the collagen 1, α-SMA, PINK1, parkin, P-parkin, and TOM20 expression in the primary HSCs. (a1) Columns represent the gray-scale value of collagen 1 expression. (a2) Columns represent the gray-scale value of the α-SMA expression. (a3) Columns represent the gray-scale value of PINK1 expression. (a4) Columns represent the gray-scale value of parkin expression. (a5) Columns represent the gray-scale value of P-parkin expression. (a6) Columns represent the gray-scale value of TOM20 expression. Data show the mean ± standard deviation. Experiments were repeated three times. *P < 0.05, and **P < 0.01.

**Figure 3:**
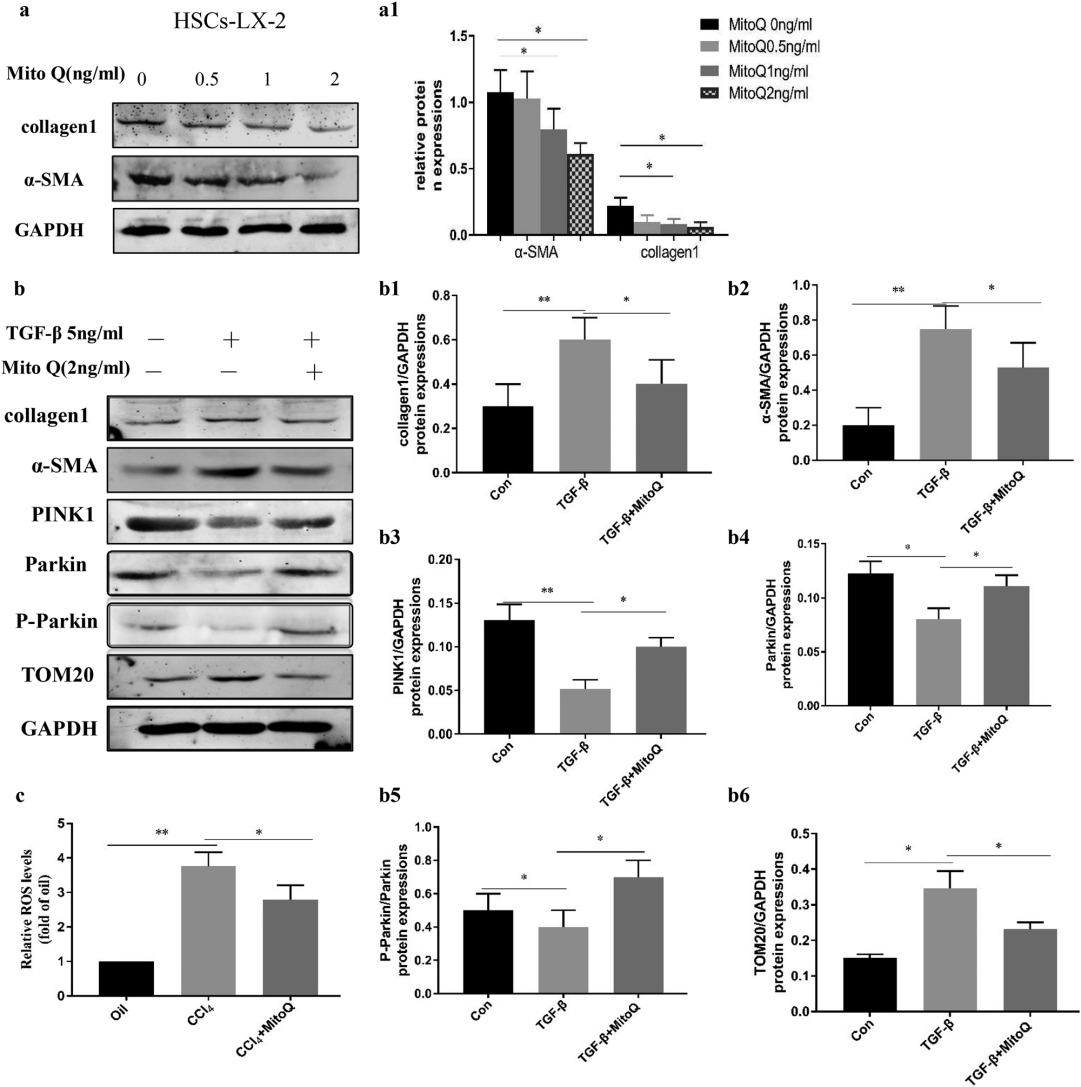
Mitophagy decreases with the LX-2 activation, and MitoQ enhances mitophagy and reduces ROS levels. (a) TGB-β to activate the LX-2 *in vitro*, Western blot analysis showing collagen 1 and α-SMA expression in LX-2 cells treated with different concentrations of MitoQ. (a1) Columns represent the gray-scale value of α-SMA and collagen 1 expression. Data show the mean ± standard deviation. Experiments were repeated three times. *P < 0.05. (b) Western blot analysis showing collagen 1, α-SMA, PINK1, parkin, P-parkin, and TOM20 expression with TGF-β stimulation and MitoQ intervention in LX-2 cells. (b1) Columns represent the gray-scale value of collagen 1 expression. (b2) Columns represent the gray-scale value of the α-SMA expression. (b3) Columns represent the gray-scale value of the PINK1 expression. (b4) Columns represent the gray-scale value of parkin expression. (b5) Columns represent the gray-scale value of P-parkin expression. (b6) Columns represent the gray-scale value of TOM20 expression. Data show the mean ± standard deviation. Experiments were repeated three times. *P < 0.05, and **P < 0.01. (c) ROS probe was used to detect the levels of ROS in LX-2 cells, *P < 0.05, and **P < 0.01.

PINK1 accumulates selectively on dysfunctional mitochondria, recruits parkin to the mitochondria, and activates it by phosphorylation of serine 65 on parkin. The E3 ubiquitin ligase activity of parkin targets mitochondria for degradation by autophagy and leads to mitochondrial protein TOM20 degradation [4]. Hence, PINK1, parkin, P-parkin, and TOM20 are important proteins involved in mitophagy. To study mitophagy during the activation of HSCs, primary HSCs were extracted from animals to determine the expression of mitophagy-related proteins. Our data demonstrated that the expression of α-SMA and collagen 1 (HSC activation marker proteins) was higher in the model group compared with the control group. Interestingly, expression of mitophagy-related proteins PINK1, parkin, and P-parkin was reduced, and TOM20 expression was increased in the primary HSCs of the model group, while MitoQ reverted this change (Figure 2a and a1–6). Furthermore, we used TGB-β to activate the HSC line LX-2 *in vitro*. The expression levels of α-SMA and collagen 1 were downregulated with increasing MitoQ concentrations in the human LX-2 cells (Figure 3a and a1). TGF-β stimulated LX-2 cells to mediate downregulation of mitophagy-related proteins PINK1, parkin, and P-parkin and increased the accumulation of α-SMA, collagen 1, and TOM20. However, MitoQ intervention increased the PINK1, parkin, and P-parkin protein expression and reduced α-SMA, collagen 1, and TOM20 expression (Figure 3b and b1–6). ROS levels were increased after TGF-β stimulation of LX-2 cells, while MitoQ intervention reduced the ROS levels (Figure 3c). Taken together, our data indicated that mitophagy was decreased with the activation of HSCs, while MitoQ enhanced the mitophagy activity of the PINK1/parkin pathway, reduced intracellular ROS levels, and inhibited LX-2 activation.

### 3.3 MitoQ inhibits HSC activation through enhancing mitophagy

**Figure 4:**
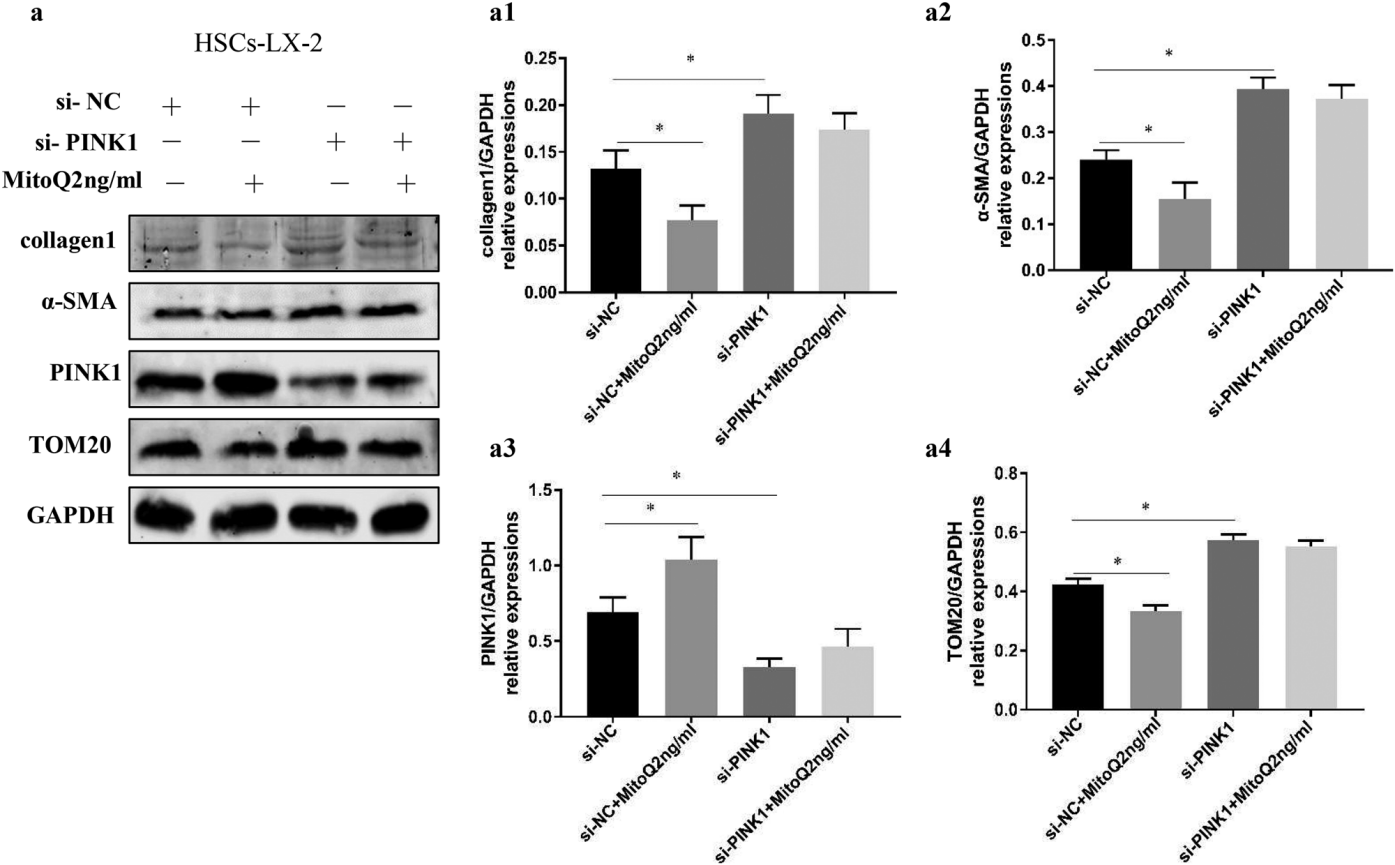
MitoQ inhibits HSC activation by enhancing mitophagy. (a) LX-2 cells stimulated by MitoQ showed reduced α-SMA, collagen 1, and TOM20 expression (si-NC vs si-NC + MitoQ). The PINK1 knockdown to inhibit mitophagy increased the expression of α-SMA, collagen 1, and TOM20 expression in the LX-2 cells (si-NC vs si-PINK1), but the expression of α-SMA, collagen1, and TOM20 were not affected by stimulation with MitoQ (si-PINK1 vs si-PINK1 + MitoQ). (a1) Columns represent the gray-scale value of collagen 1 expression. (a2) Columns represent the gray-scale value of the α-SMA expression. (a3) Columns represent the gray-scale value of the PINK1 expression. (a4) Columns represent the gray-scale value of TOM20 expression. Data show the mean ± standard deviation. Experiments were repeated three times. *P < 0.05.

To further test whether MitoQ inhibited HSC activation by enhancing mitophagy, PINK1 was knocked down by siRNA in LX-2 cells. The PINK1 knockdown led to an increase in α-SMA, collagen 1, and TOM20 expression, suggesting that inhibition of PINK1/parkin-induced mitophagy enhanced HSC activation and increased the number of residual mitochondria. However, after knocking down PINK1, the application of MitoQ did not reduce α-SMA and collagen 1 expression or lead to changes in TOM20 expression. These findings suggested that MitoQ reduced HSC activation by enhancing the PINK1/parkin-mediated mitophagy pathway (Figure 4a and a1–4).

## 4 Discussion

PINK1/parkin-mediated mitophagy plays an important role in autophagy-dependent mitochondrial degradation in mammals. MitoQ is an antioxidant that specifically targets mitochondria. However, research on MitoQ and mitophagy in the process of liver fibrosis has been limited. In this study, we found that PINK1/parkin-mediated mitophagy was downregulated during the activation of HSCs and development of hepatic fibrosis, while MitoQ enhanced mitophagy and reduced ROS levels in the HSCs and reduced liver fibrosis.

Liver fibrosis in mice is usually induced by CCl4 administration or bile duct ligation (BDL) [30,31]. This study established a CCl_4_-induced hepatic fibrosis mouse model and used the Ishak score to assess the degree of fibrosis. We observed a reduced Ishak score in the MitoQ intervention group, indicating successful modeling of hepatic fibrosis, while MitoQ reduced hepatic fibrosis.

Recent studies have shown that mitophagy may participate in the pathogenesis of various liver diseases, such as viral hepatitis, liver injury, hepatocellular carcinoma, liver steatosis/fatty liver disease, and hepatic fibrosis [32]. However, studies investigating the role of mitophagy in liver fibrosis have mainly focused on hepatocytes and Kupffer cells (KCs). Melatonin protects against liver fibrosis via upregulation of mitophagy [33]. TIM‐4 interference in the KCs inhibits Akt1‐mediated ROS production, resulting in the suppression of PINK1, parkin, and LC3‐II/I activation, reduction of TGF‐β1, and attenuation of fibrosis development [34]. One study mentioned that resveratrol induces mitophagy in HSCs [35], and mitophagy was enhanced in a hepatic fibrosis-reversal model [13]. However, the effect of mitophagy on HSC survival and hepatic fibrosis was unclear. We found that PINK1/parkin-mediated mitophagy was downregulated during the activation of the HSCs, which is consistent with the previous studies [13,36].

Mitochondria-targeted antioxidant mitoquidone improves liver inflammation and fibrosis in rats with cirrhosis by reducing hepatic oxidative stress, preventing apoptosis, and promoting the removal of dysfunctional mitochondria. However, the research focused on hepatocytes [37]. One study reported that mitoquidone deactivates human and rat HSCs and reduces portal hypertension in rats with cirrhosis through decreased oxidative stress levels [19]. However, the relationship between MitoQ and mitophagy in HSCs has been unknown. MitoQ exerts beneficial effects on tubular injury in diabetic kidney disease via Nrf2/PINK1-mediated mitophagy [25]. Thus, we speculated that MitoQ enhanced PINK1/parkin-induced mitophagy in HSCs to reduce liver fibrosis. In this study, we found that MitoQ reduced the ROS levels, upregulated mitophagy, and deactivated LX-2 cells and primary HSCs; this was reflected in increased PINK1, parkin, and P-parkin expression, and in reduced TOM20, α-SMA, and collagen 1 expression after the MitoQ intervention in primary HSCs and LX-2 cells. This study demonstrated that MitoQ can affect HSC activation by regulating mitophagy, which has not been reported in this context previously.

## 5 Conclusion

In summary, this study demonstrated that mitophagy is weakened during HSC activation. We propose a new mechanism that MitoQ inhibits HSC activation and plays an antifibrotic role through regulating PINK1/parkin-mediated mitophagy and ROS production.
